# Ospemifene displays broad-spectrum synergistic interactions with itraconazole through potent interference with fungal efflux activities

**DOI:** 10.1038/s41598-020-62976-y

**Published:** 2020-04-08

**Authors:** Hassan E. Eldesouky, Ehab A. Salama, Tony R. Hazbun, Abdelrahman S. Mayhoub, Mohamed N. Seleem

**Affiliations:** 1 Department of Comparative Pathobiology, College of Veterinary Medicine, Purdue University, West Lafayette, IN 47907 USA; 2 Department of Medicinal Chemistry and Molecular Pharmacology, College of Pharmacy, Purdue University, West Lafayette, Indiana 47907 USA; 3 Bindley Bioscience Center, Purdue University, West Lafayette, Indiana 47906 USA; 4 University of Science and Technology, Nanoscience Program, Zewail City of Science and Technology, October Gardens, 6th of October, Giza, 12578 Egypt; 5 Purdue Institute of Inflammation, Immunology, and Infectious Disease, Purdue University, West Lafayette, IN 47907 USA

**Keywords:** Antifungal agents, Fungal pathogenesis

## Abstract

Azole antifungals are vital therapeutic options for treating invasive mycotic infections. However, the emergence of azole-resistant isolates combined with limited therapeutic options presents a growing challenge in medical mycology. To address this issue, we utilized microdilution checkerboard assays to evaluate nine stilbene compounds for their ability to interact synergistically with azole drugs, particularly against azole-resistant fungal isolates. Ospemifene displayed the most potent azole chemosensitizing activity, and its combination with itraconazole displayed broad-spectrum synergistic interactions against *Candida albicans*, *Candida auris*, *Cryptococcus neoformans*, and *Aspergillus fumigatus* (ΣFICI = 0.05–0.50). Additionally, in a *Caenorhabditis elegans* infection model, the ospemifene-itraconazole combination significantly reduced fungal CFU burdens in infected nematodes by ~75–96%. Nile Red efflux assays and RT-qPCR analysis suggest ospemifene interferes directly with fungal efflux systems, thus permitting entry of azole drugs into fungal cells. This study identifies ospemifene as a novel antifungal adjuvant that augments the antifungal activity of itraconazole against a broad range of fungal pathogens.

## Introduction

Invasive mycotic infections are life-threatening medical conditions that claim the lives of more than 1.5 million patients worldwide^1,2^. Invasive mycoses mainly affects immunocompromised individuals, such as HIV patients and organ transplant recipients^3,4^. The majority of invasive fungal infections are attributed to *Candida*, *Cryptococcus*, and *Aspergillus* species^1^. *Candida* species are the most common fungal pathogen that infects humans and are associated with more than 200,000 fatalities worldwide each year^5,6^. In the USA, *Candida* species are the fourth-leading cause of nosocomial bloodstream infections^7,8^. Invasive candidiasis is mainly attributed to five species, *C. albicans*, *C. glabrata*, *C. parapsilosis*, *C. tropicalis*, and *C. Krusei*^9,10^. However, *C. auris* is an emerging pathogen of a global public health concern due to its unique resistance profile to multiple antifungal drugs and associated high mortality rates (~30–70%)^11,12^. Recently, the U.S. Centers for Disease Control and Prevention (CDC) has labeled *C. auris* as an urgent threat that requires immediate action^13^. Likewise, *Cryptococcus* species, especially *C. neoformans*, are major fungal pathogens and a leading cause of death in AIDS patients, causing more than 600,000 deaths worldwide annually^14^. Another medically important fungal pathogen is *Aspergillus fumigatus* which is the main cause of recalcitrant invasive aspergillosis and also is associated with devastatingly high mortality rates (up to 95%)^1^.

The high mortality rates linked to invasive mycotic infections are mainly attributed to resistance to current antifungal drugs, lack of rapid diagnostics, and limited therapeutic options^15–19^. Only three main drug classes (polyenes, echinocandins, and azoles) are available to treat systemic mycoses^17^. Azoles are the only orally-bioavailable antifungal drugs that possess broad-spectrum antifungal activity with limited side effects, especially compared to polyenes^20^. Thus, azoles are the most commonly prescribed antifungal drugs for treating a wide variety of fungal infections^21^. Unfortunately, the extensive use of azoles has been linked to the increased frequency of azole-resistant fungal infections^22–24^.

Given the dearth of current antifungal drugs, identifying molecules capable of enhancing the antifungal activity of azole drugs, especially against resistant fungal species, is an appealing alternative drug discovery approach. To this effect, we previously evaluated a library of FDA-approved drugs and clinical molecules for their ability to re-sensitize azole-resistant *C. albicans* to the effect of fluconazole. Multiple stilbene derivatives such as tamoxifen, diethylstilbestrol, and hexestrol were found able to interact synergistically with fluconazole. Consistent with our results, previous studies have reported tamoxifen’s ability to re-sensitize *C. neoformans* to the effect of azole drugs, both *in vitro* and *in vivo* in a murine model^25–27^. However, the azole chemosensitizing activity of other stilbene derivatives remains unexplored. Additionally, the interactions of stilbene derivatives with newer azoles, and their activities against emerging multidrug-resistant fungal species such as *C. auris*, have not been investigated.

In this study, the antifungal activity of nine stilbene derivatives was tested, with or without fluconazole, against an azole-resistant *C. albicans* isolate. Ospemifene, an oral estrogen receptor modulator, displayed the most potent synergistic activity with fluconazole and was further explored in combination with different azole drugs against a panel of fungal pathogens including *C. albicans*, *C. glabrata*, *C. auris*, *C. neoformans*, and *A. fumigatus*. Furthermore, we assessed the effect of ospemifene on the efflux activity of *Candida* species to identify a mechanism for ospemifene’s azole chemosensitizing activity.

## Results

### Fluconazole chemosensitizing activity of stilbene derivatives

**Figure 1:**
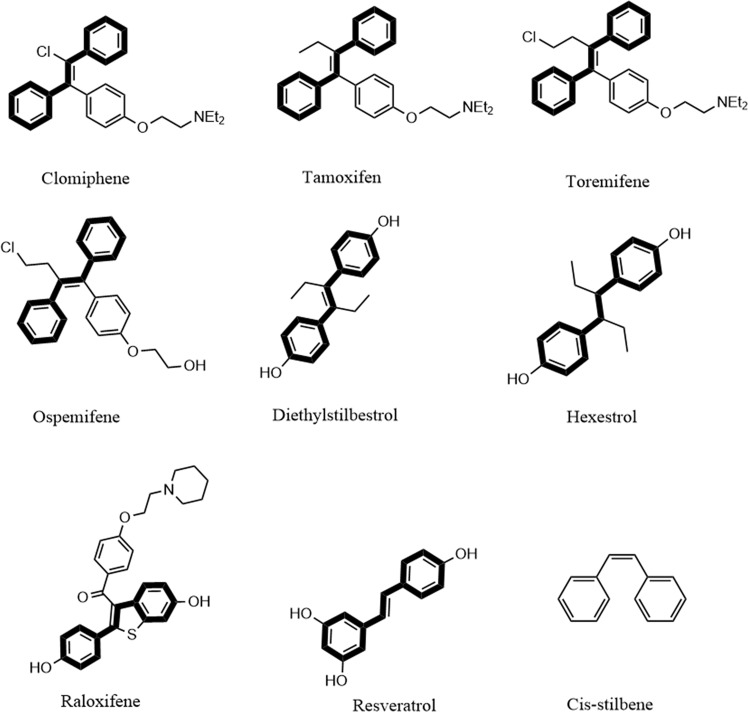
Chemical structures of stilbene compounds. The trans-stilbene scaffold is highlighted as bold black bonds.

In a previous study, we screened the Pharmakon drug library to identify novel adjuvants that would enhance fluconazole’s antifungal activity against an azole-resistant *C. albicans* isolate. Our initial screen revealed three stilbene derivatives (tamoxifen, hexestrol, and diethylstilbestrol) that were able to interact synergistically with fluconazole. This result encouraged us to further evaluate other stilbene compounds for their ability to enhance the activity of azole antifungal drugs. We investigated the fluconazole chemosensitizing activity of six additional stilbene compounds, namely clomiphene, toremifene, raloxifene, ospemifene, resveratrol, and cis-stilbene (Fig. 1). As presented in Table 1, diethylstilbestrol and hexestrol exhibited a moderate synergistic relationship with fluconazole against *C. albicans* NR-29448 (ΣFICI = 0.50). Clomiphene, toremifene, tamoxifen, and raloxifene displayed more potent fluconazole chemosensitizing activity (ΣFICI = 0.26). Interestingly, ospemifene exhibited the most potent synergistic interaction with fluconazole against *C. albicans* NR-29448 (ΣFICI = 0.02). resveratrol displayed an additive effect when combined with fluconazole (ΣFICI = 0.53) while cis-stilbene exhibited an indifferent relationship with fluconazole against *C. albicans* NR-29448 (ΣFICI = 1.01).

**Table 1:** Effect of different combinations of stilbene derivatives with fluconazole (FLC) against *C. albicans* NR-29448.

Stilbenes drugs	MIC (µg/ml)				ΣFICI^a^	Interaction
	FLC		Stilbenes drugs			
	Alone	Combined	Alone	Combined		
Clomiphene	256	2	8	2	0.26	SYN
Tamoxifen		2	16	4	0.26	SYN
Toremifene		2	8	2	0.26	SYN
Hexestrol		1	8	4	0.50	SYN
Diethylstilbestrol		1	4	2	0.50	SYN
Ospemifene		2	>256	2	0.02	SYN
Raloxifene		2	64	16	0.26	SYN
Resveratrol		8	> 256	128	0.53	ADD
cis-stilbene		2	> 256	> 256	1.01	IND

^a^ΣFICI (fractional inhibitory concentration index) values, rounded to the nearest two decimal places, were used to measure the interaction between the tested combinations. ΣFICI interpretation corresponded to the following definitions: synergistic (SYN), ΣFICI ≤ 0.50; additive (ADD), ΣFICI > 0.50 and ≤ 1; and indifference (IND), ΣFICI > 1 and ≤ 4.

### Effect of ospemifene on the antifungal activity of other azole drugs

As ospemifene exhibited the most potent fluconazole chemosensitizing activity amongst the stilbene derivatives tested, ospemifene was selected for further investigation. The antifungal activity of ospemifene, with and without different azole drugs, was evaluated against a panel of 23 fungal isolates including strains of *C. albicans, C. glabrata, C. auris, C. neoformans*, and *A. fumigatus*. Ospemifene exhibited a synergistic relationship when combined with fluconazole or voriconazole against 22% and 26% of the isolates tested, respectively (Supplementary Table 2 and Supplementary Table 3). However, as shown in Table 2, ospemifene exhibited broad-spectrum synergistic interactions with itraconazole against all tested isolates, except for *C. glabrata*, resulting in a reduction in the minimum inhibitory concentration (MIC) of itraconazole by 2- to 64-fold (ΣFICI = 0.05–0.5).

**Table 2:** Effect of ospemifene-itraconazole (ITC) combination against different fungal strains.

Fungal Strains	MIC (µg/ml)				ΣFICI^a^	Interaction
	ITC^*^		Ospemifene			
	Alone	Combined	Alone	Combined		
*C. albicans* SC5314	0.125	0.0312	>256	2	0.26	SYN
*C. albicans* NR-29448	4^*^	0.0625		8	0.05	SYN
*C. albicans* NR-29437	0.5	0.06		4	0.14	SYN
*C. albicans* ATCC MYA-573	0.5	0.25		1	0.50	SYN
*C. albicans* TWO7241	0.5	0.125		0.5	0.25	SYN
*C. albicans* TWO7243	1^*^	0.5		1	0.50	SYN
*C. albicans* SC-TAC1^G980E^	0.5	0.25		1	0.50	SYN
*C. albicans* SC-MRR1^P683S^	0.25	0.062		4	0.26	SYN
*C. glabrata* ATCC 66032	0.5	0.5		4	1.02	IND
*C. glabrata* ATCC MYA-2950	0.5	0.5		4	1.02	IND
*C. glabrata* ATCC 2001	0.5	0.5		4	1.02	IND
*C. glabrata* HM-1123	0.5	0.5		4	1.02	IND
*C. auris* 385	1^*^	0.25		4	0.27	SYN
*C. auris* 386	0.5	0.125		4	0.27	SYN
*C. auris* 388	1^*^	0.25		4	0.27	SYN
*C. auris* 389	1^*^	0.25		4	0.27	SYN
*C. auris* 390	1^*^	0.125		4	0.14	SYN
*C. neoformans* NR-41291	0.25	0.062		4	0.26	SYN
*C. neoformans* NR-41295	0.5	0.25		1	0.50	SYN
*C. neoformans* NR-41298	0.5	0.125		2	0.26	SYN
*A. fumigatus* NR-35304	0.5	0.25		0.5	0.50	SYN
*A. fumigatus* NR-35312	0.5	0.25		2	0.50	SYN
*A. fumigatus* NR-35302	0.5	0.25		1	0.50	SYN

^a^ΣFICI (fractional inhibitory concentration index) values, rounded to the nearest two decimal places, were used to measure the interaction between the tested combinations. ΣFICI interpretation corresponded to the following definitions: synergistic (SYN), ΣFICI ≤ 0.50; additive (ADD), ΣFICI > 0.50 and ≤1; and indifference (IND), ΣFICI > 1 and ≤ 4.

^*^Indicates itraconazole resistance. MIC values of ≥ 1 µg/ml and > 2 µg/ml were selected as tentative itraconazole resistance breakpoints against yeast-like pathogens and molds, respectively^52,53^.

### Effect of the ospemifene-itraconazole combination on the growth kinetics of *Candida*, *Cryptococcus*, and *Aspergillus* species

**Figure 2:**
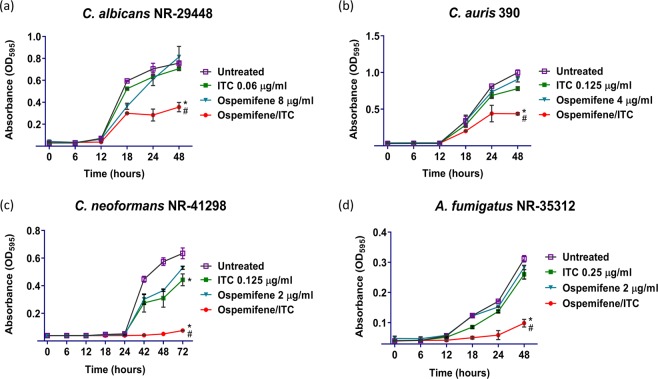
Effect of the ospemifene-itraconazole combination on the growth kinetics of different fungal species. Overnight cultures of fungal isolates were diluted to 0.5–2.5 × 10^3^ CFU/ml in RPMI 1640 medium. Cells were treated with ospemifene, itraconazole (ITC), or a combination of the two drugs at the indicated concentrations. Cells were incubated at 35 °C for 48–72 h, and OD_595_ values were measured at different time points (0, 6, 12, 18, 24, 36 and 48 h). *Indicates statistical significance relative to the treated control while (#) indicates statistical significance (*P* < 0.05) relative to individual treatments with ospemifene or itraconazole. The statistical significance was determined by multiple t-tests using the Holm-Sidak statistical method for multiple comparisons.

To confirm the broad-spectrum itraconazole chemosensitizing activity of ospemifene, we evaluated the effect of the ospemifene-itraconazole combination on the growth kinetics of four fungal isolates most susceptible to the drug combination. As shown in Fig. 2, the ospemifene-itraconazole combination (at concentrations identified from the previous microdilution checkerboard assay) significantly reduced the growth of *C. albicans* NR-29448 (panel a), *C. auris* 390 (panel b), *C. neoformans* NR-41298 (panel c), and *A. fumigatus* NR-35312 (panel d) compared to treatment with either ospemifene or itraconazole alone.

### Ospemifene interferes with fungal ABC and MFS efflux pumps

**Figure 3:**
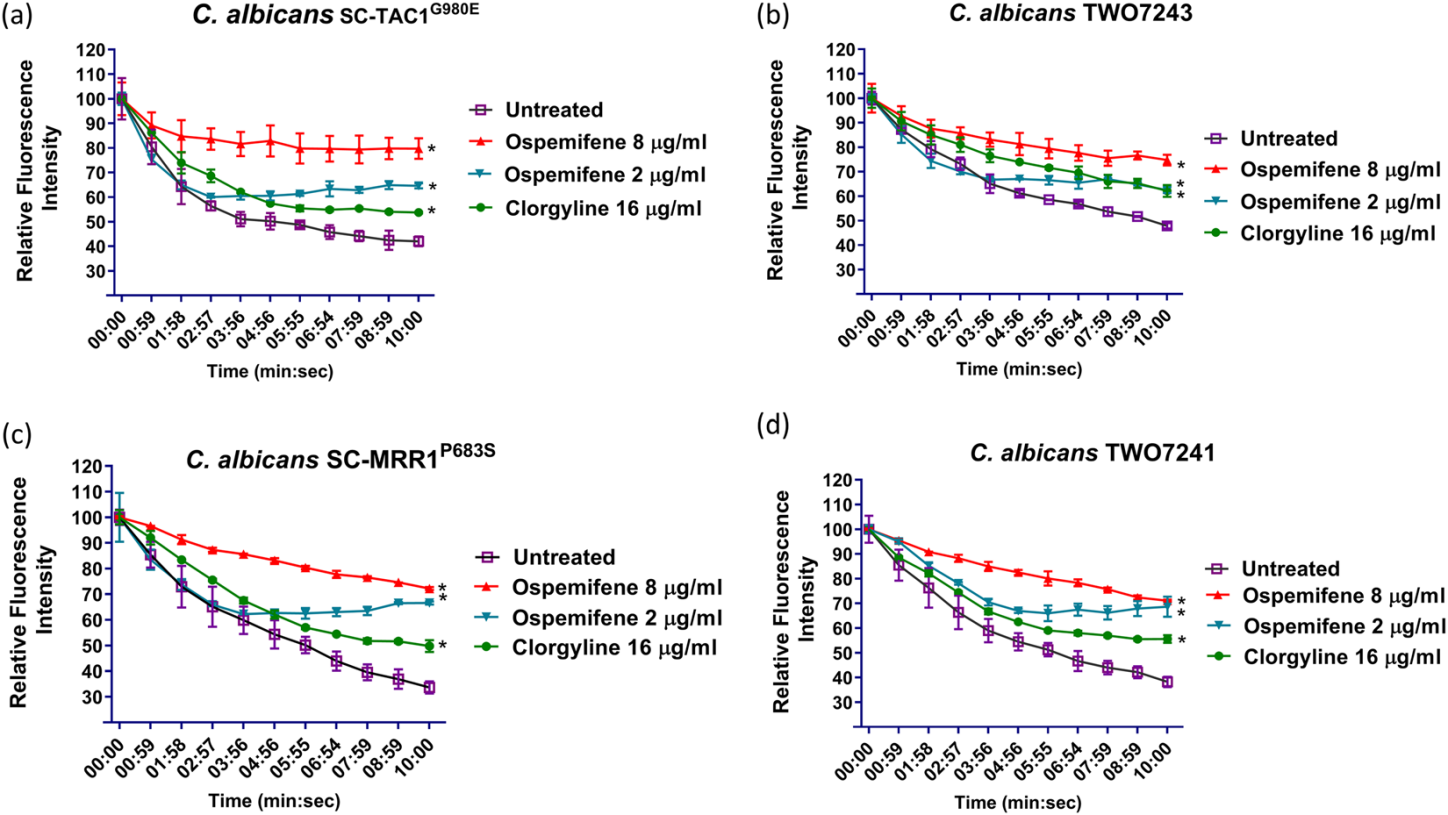
Effect of ospemifene on Nile red efflux by different efflux hyperactive *Candida* strains. Effect of ospemifene on Nile red efflux in ABC-efflux hyperactive *C. albicans* strains (**a**) SC-TAC1^G980E^ and (**b**) TWO7243, and MFS efflux hyperactive *C. albicans* strains (**c**) SC-MRR1^P683S^ and (**d**) TWO7241. Energy-depleted cells were loaded with Nile red (7.5 µM) and were subsequently treated with ospemifene (2 and 8 µg/ml) or clorgyline (16 µg/ml). Efflux was initiated by adding glucose (40 mM) to all treatment groups. The Nile red fluorescence intensity was then monitored over 10 minutes and is expressed as the percentage of change in the fluorescence intensity. *Indicates a statistically significant difference in the fluorescence intensity of the treated cells relative to the untreated control (*P* < 0.05), as determined by multiple t-tests using the Holm-Sidak test for multiple comparisons.

Nile red efflux assays were used to study the impact of ospemifene on the efflux activity the ATP-binding cassette (ABC) and the major facilitator superfamily (MFS) membrane transporters expressed by *Candida* species. ABC and MFS transporters in fungi are known to play an important role in conferring resistance to azole antifungals^28–30^. As shown in Fig. 3, ospemifene, in a concentration-dependent manner, significantly interfered with Nile red efflux from the ABC efflux-activated strains (SC-TAC1^G980E^ and TWO7243) and also from the MFS efflux-activated strains (SC-MRR1^P683S^ and TWO7241) compared to the untreated control. Interestingly, the ability of ospemifene to interfere with Nile red efflux was found to be more significant than clorgyline, a known fungal efflux pump inhibitor^31^.

### Effect of ospemifene on the transcription levels of azole resistance-related efflux genes

**Figure 4:**
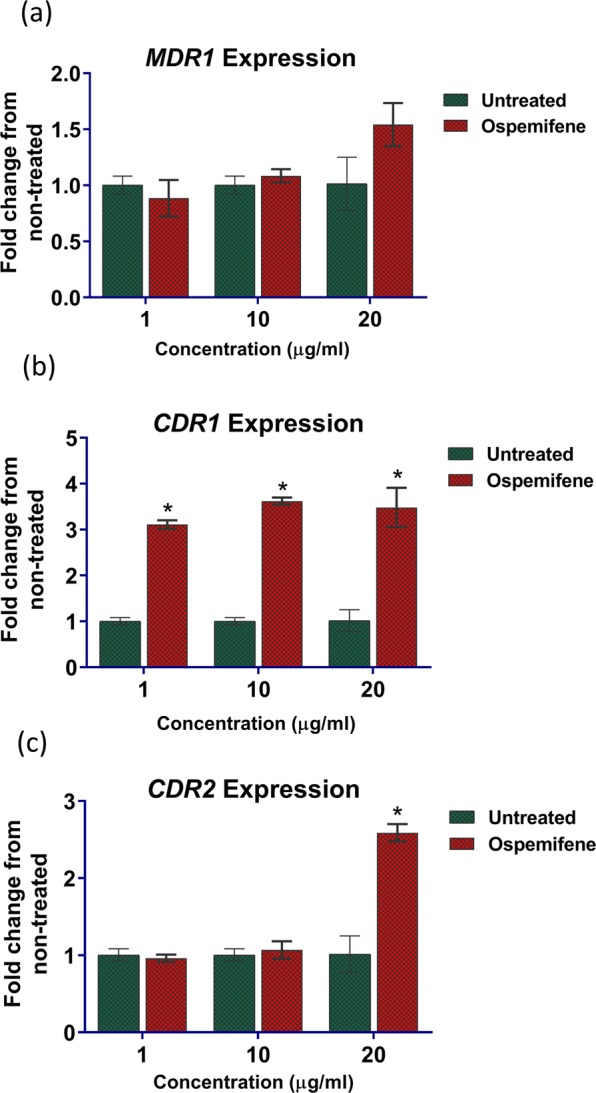
Effect of ospemifene on the expression of azole resistance-related efflux genes. *C. albicans* SC5314 was treated with either DMSO or ospemifene (1, 10, 20 µg/ml) for 3 h in RPMI 1640 and then harvested. The expression of (**a**) *CDR1*, (**b**) *CDR2*, and (**c**) *MDR1* was determined by quantitative RT-PCR. Bars display the mean fold-change for ospemifene-treated cells relative to untreated cells. Error bars represent standard deviation values from three biological replicates. *Indicates a statistically significant difference between the ospemifene treatment relative to the untreated control (*P* < 0.05), as determined by multiple t-tests using the Holm-Sidak test for multiple comparisons.

The effect of ospemifene on the mRNA levels of genes related to efflux pump expression (namely, *CDR1*, *CDR2*, and *MDR1*) was investigated using RT-qPCR. As shown in Fig. 4, ospemifene treatment at only 1 µg/ml, resulted in a significant increase (by 3-fold) in the mRNA levels of *CDR1* relative to the untreated control. However, a significant increase (by 2.6-fold) in the transcription level of *CDR2* was only observed at a higher concentration of ospemifene (20 µg/ml). Additionally, a statistically insignificant increase (~1.5-fold) in the expression level of the MFS efflux gene *MDR1* was observed with ospemifene treatment at 20 µg/ml.

### The ospemifene-itraconazole combination reduces fungal burden in infected *Caenorhabditis elegans*

**Figure 5:**
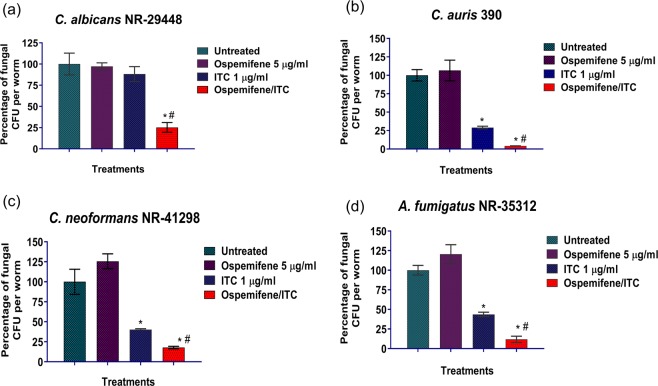
*In vivo* efficacy of the ospemifene-itraconazole combination in reducing fungal burden in infected *C. elegans*. *C. elegans* strain AU37 genotype [glp-4(bn2) I; sek-1(km4) X], was infected with (**a**) *C. albicans* NR-29448, (**b**) *C. auris* 390, (**c**) *C. neoformans* NR-41298, or (**d**) *A. fumigatus* NR-35312, using an inoculum size of ~5 × 10^7^ CFU/ml for three hours at room temperature. Infected nematodes were washed with PBS and then treated with the ospemifene (5 µg/ml)-itraconazole (1 µg/ml) combination at the respective concentration. Treatment with PBS, ospemifene, or itraconazole (ITC) alone served as controls. After 24 h of treatment, worms were lysed to determine the fungal burden (CFU/worm). *Indicates a statistically significant difference between each treatment compared to the untreated control (*P* < 0.05). (#) indicates a statistically significant difference between the ospemifene-itraconazole combination relative to treatment with either ospemifene or itraconazole (ITC) alone (*P* < 0.05), as determined by a one-way ANOVA using post-hoc Dunnet’s test for multiple comparisons.

To further support our *in vitro* results, the azole chemosensitizing activity of ospemifene was investigated in a *C. elegans* infection model. First, we assessed the toxicity of ospemifene in non-infected *C. elegans* nematodes and found that ospemifene can be used safely at concentrations up to 40 µg/ml (Supplementary Fig. 1). Next, a subtoxic concentration of ospemifene (5 µg/ml) was used in combination with itraconazole (1 µg/ml) to treat *C. elegans* nematodes infected with either *C. albicans*, *C. auris*, *C. neoformans*, or *A. fumigatus*. As shown in Fig. 5, the combination of ospemifene and itraconazole was able to significantly reduce the mean fungal CFU by ~75%, 96%, 82%, and 88% in nematodes infected with either *C. albicans* NR-29448 (Fig. 5a), *C. auris* 390 (Fig. 5b), *C. neoformans* NR-41298 (Fig. 5c), and *A. fumigatus* NR-35312 (Fig. 5d), respectively. However, itraconazole alone, at 1 µg/ml, was able to reduce fungal burden by ~39%, 71%, 60%, and 56% against *C. albicans* NR-29448, *C. auris* 390, *C. neoformans* NR-41298, and *A. fumigatus* NR-35312, respectively. As expected, ospemifene alone, at 5 µg/ml, failed to reduce fungal CFU in the infected nematodes against all four fungal strains tested.

## Discussion

Azole antifungals are vital therapeutics for the treatment of systemic fungal infections. Unfortunately, the emergence of clinical fungal isolates exhibiting resistance to azole antifungals has limited their effectiveness. To ameliorate this challenge, we were interested in exploring new adjuvants capable of enhancing or restoring the antifungal activity of azole antifungals, especially against azole-resistant fungal pathogens. To this end, we investigated the azole chemosensitizing activity of nine stilbene compounds. Microdilution checkerboard assays revealed prominent fluconazole chemosensitizing activity was only achieved by trans-stilbene derivatives, suggesting that the trans-stilbene configuration is important for the fluconazole chemosensitizing effect observed. The absence of any detectable fluconazole chemosensitizing activity for compounds possessing the cis-stilbene structure further strengthens the premise that the trans-configuration is critical for the fluconazole chemosensitizing activity observed with stilbene derivatives.

Notably, the fluconazole chemosensitizing activity of ospemifene was found to be superior to the other pharmacologically-related estrogen receptor antagonists. Therefore, ospemifene was evaluated further to gauge its spectrum of azole chemosensitizing activity against a panel of clinically-relevant fungal pathogens. Interestingly, the ospemifene-itraconazole combination displayed broad-spectrum synergistic activity against strains of *C. albicans*, multidrug-resistant *C. auris, C. neoformans*, and *A. fumigatus*. Importantly, ospemifene (at 4 µg/ml) was able to sensitize multidrug-resistant *C. auris* to the antifungal effect of itraconazole (reducing its MIC by 4- to 8-fold). The synergistic relationship between ospemifene and itraconazole was further validated *in vivo* in a *C. elegans* infection model. Ospemifene, at only 5 µg/ml, synergistically interacted with itraconazole to significantly reduce the burden of *C. albicans, C. auris*, *C. neoformans*, and *A. fumigatus* in infected nematodes. It should be noted that itraconazole is known to exert a fungistatic activity against yeast-like pathogens and a fungicidal effect against molds^32^. Our data indicate that ospemifene didn’t alter the fungistatic nature of itraconazole against the yeast pathogens *C. albicans* and *C. auris*. However, the ospemifene-itraconazole combination exhibited fungicidal activity (~4-log reduction in CFU) against *C. neoformans* (Supplementary Fig. 2). This observation suggests a potential clinical significance of ospemifene as an adjunct therapy for treating cryptococcal infections, especially in immunocompromised patients where fungicidal drugs are desperately needed. However, further *in vivo* assessment in other animal models is required to evaluate ospemifene as a potential adjuvant with azoles in the treatment of fungal infections.

It is important to note that estrogen receptor modulators have been reported to possess intrinsic antifungal activities and also been shown to interact synergistically with fluconazole^27,33^. The antifungal activity of estrogen receptor compounds is believed to be mediated through interference with the fungal calcineurin pathway^26,33^. The existence of an alkylamino group attached to the aromatic system of the triphenylethylene core was reported to be essential for the calcineurin inhibitory activities of estrogen receptor modulators^34^. However, ospemifene lacks this feature and thus its azole chemosensitizing activity is unlikely to be mediated by the interference of the calcineurin pathway. To this end, we observed that ospemifene is capable of sensitizing efflux-hyperactive *Candida* strains (SC-TAC1^G980E^, TWO7243, SC-MRR1^P683S^, and TWO7241) to the antifungal effect of itraconazole. Strains SC-TAC1^G980E^, TWO7243 are known to have increased mRNA levels of ABC-type membrane transporters, while strains SC-MRR1^P683S^, and TWO7241 exhibit upregulated expression of MFS-type membrane transporters^35–37^. Thus, we postulated that the mechanism by which ospemifene enhanced the sensitivity of fungal species to the effect of itraconazole was by interfering with the efflux activity of fungal membrane transporters. We utilized Nile red efflux assays to examine the effect of ospemifene on the efflux activity of *Candida*. Our results indicate that ospemifene significantly hindered the efflux of Nile red from several efflux-hyperactive strains (irrespective if they were ABC- or MFS-mediated). Interestingly, the efflux inhibitory activity of ospemifene was found to be superior to clorgyline, a known fungal efflux pump inhibitor^31^. To ensure that ospemifene can interfere specifically with the drug transporters pertinent to azole resistance, we conducted efflux assays using recombinant *Saccharomyces cerevisiae* strains overexpressing individual transporters, *CDR1*, *CDR2*, or *MDR1*, from the human pathogen *Candida albicans*^31,38^. Further corroborating our previous results, ospemifene treatment resulted in a significant increase in Nile red fluorescence intensity in all recombinant strains compared to the untreated control (Supplementary Fig. 3). We also observed that ospemifene treatment increased the mRNA expression levels of azole resistance-related efflux genes, particularly the ABC efflux gene *CDR1*, suggesting that ospemifene may interfere directly with fungal efflux function. Similar increases in the expression levels of efflux genes were observed with clorgyline, a known broad-spectrum efflux inhibitor (Supplementary Fig. 4)^31^. Of note, milbemycin oxime, another known fungal efflux pump inhibitor, was reported to induce the expression of MFS efflux transporters in *C. albicans*^39^. These findings suggest that ospemifene may directly interfere with the efflux of azole drugs probably by acting as a more favorable substrate for efflux than azoles.

It should be mentioned that the clinical significance of ospemifene as a potential antifungal adjuvant therapy stems from its ability to exert a broad-spectrum chemosensitizing activity at clinically-applicable concentrations. According to a report issued by European Medicines Agency, ospemifene was shown to achieve a maximum human serum concentration of 2 µM (0.785 µg/ml) and up to 24 µg/ml in the intestinal tract at normally indicated dosing^40^. As presented in Table 2, except for *C. glabrata*, ospemifene was able to sensitize all fungal isolates tested to itraconazole at concentrations that can be easily achieved in the human gut, while ~37% of isolates (seven strains) were sensitized at concentrations ≤1 µg/ml, which is close to ospemifene’s maximum serum concentration. These results are suggestive of the potential of repurposing ospemifene as an adjunct therapy for controlling invasive fungal infections, especially in intestinal mycoses. Of note, serious negative complications such as endometrium cancer and thromboembolic effects have been reported with long-term use of estrogen modulating therapeutics^41^. However, ospemifene represents a new class of selective estrogen receptor modulators that was shown to be well-tolerated and the undesired effects on the endometrium or blood coagulation were not reported even with long-term use^41^.

In conclusion, this study presents trans-stilbene derivatives as potential adjuvants to enhance the antifungal activity of azole drugs. A preliminary Structural-Activity-Relationship (SAR) analysis of stilbene derivatives revealed that the trans-configuration of the stilbene scaffold is vital for azole chemosensitizing activity. Ospemifene was found to be the most effective azole chemosensitizing agent and exhibited a broad-spectrum synergistic relationship with itraconazole against multiple fungal species both *in vitro* and in a *C. elegans* infection model. More importantly, the ospemifene-itraconazole combination was effective against emerging multidrug-resistant *C. auris* isolates and exerted a fungicidal effect against *C. neoformans*. Our preliminary mechanistic studies indicate ospemifene is a potent inhibitor of fungal efflux pumps, which may explain ospemifene’s ability to enhance the antifungal activity of azole drugs.

## Materials and methods

### Fungal strains and culture reagents

Sources and descriptions of the fungal strains used in this study are provided in Supplementary Table 1. Yeast extract-peptone-dextrose (YPD) agar and broth were purchased from Becton and Dickinson Company (Franklin Lakes, NJ). 3-(N-Morpholino) propanesulfonic acid (MOPS) was purchased from Sigma-Aldrich (St. Louis, MO). RPMI 1640 powder, supplemented with glutamine and lacking NaHCO_3_, was obtained from Thermo Fisher Scientific (Waltham, MA).

### Chemicals and drugs

Clorgyline, clomiphene, toremifene, tamoxifen, raloxifene, and ospemifene were obtained from Cayman Chemicals (Ann Arbor, MI). Fluconazole was purchased from Fisher Scientific (Pittsburgh, PA). Hexestrol, 2-deoxyglucose, and cis-stilbene were purchased from Alfa Aesar (Tewksbury, MA). Nile red, itraconazole, voriconazole, and resveratrol were purchased from TCI America (Portland, OR). Glucose and diethylstilbestrol were purchased from Sigma-Aldrich (St. Louis, MO). Gentamicin sulfate was obtained from Chem-Impex International Inc. (Wood Dale, IL).

### Microdilution checkerboard assays

The interaction between stilbene derivatives and azole drugs was investigated using standard broth microdilution checkerboard assays, following previously reported protocols^42–44^. The fractional inhibitory concentration index (ΣFICI) was used to assess the relationship between the combination of azole drugs with each stilbene derivative. Synergistic (SYN) interactions were recorded when ΣFICI values were ≤0.50, additive (ADD) interactions were recorded for ΣFICI values >0.50 and ≤1, while indifference (IND) was recorded for ΣFICI values >1 and ≤4^39^.

### Growth kinetics

The impact of the ospemifene-itraconazole combination on the growth kinetics of *C. albicans* NR-29448, *C. auris* 390, *C. neoformans* NR-41298, and *A. fumigatus* NR-35312 was evaluated by turbidity measurement (using a spectrophotometer) as previously described^45^. Briefly, overnight cultures of the tested isolates were adjusted to 0.5–2.5 × 10^3^ CFU/ml in RPMI 1640 medium and incubated with ospemifene/itraconazole at concentrations selected based on the checkerboard results. Drug-free medium served as the untreated control. The fungal cultures were incubated at 35 °C and the optical densities (OD) were measured at 595 nm (OD_595_) at different time points (0, 6, 12, 18, 24, and 48 h).

### Nile red efflux assay

The impact of ospemifene as a potential fungal efflux pump inhibitor was investigated using the Nile red efflux assay, as previously described^46–48^. Briefly, exponentially grown yeast cells were incubated for 2 h at 35 °C using a rotating shaker (at 180 rpm) to induce starvation. Energy depleted cells were kept overnight on ice. Cells were then treated with Nile red (7.5 µM) for 30 minutes. Nile red-loaded cells were washed twice with PBS to remove the unbound dye. Nile red-stained cells were then transferred to 96-well plates containing serial dilutions of ospemifene or clorgyline (as a positive control). Drug-free wells served as negative control. Nile red efflux was initiated by adding glucose (40 mM) and the fluorescence intensity was measured at 485/528 nm using a SpectraMax i3x microplate reader (Molecular Devices, CA). Detection of the fluorescence intensity was started 15 seconds after glucose addition (T_0_), and Nile red fluorescence intensity was expressed as the percentage of change in the fluorescence intensity relative to the T_0_ intensity.

### Transcriptional analysis of azole resistance-associated efflux genes

The effect of ospemifene on the mRNA expression of *CDR1, CDR2, and MDR1* was evaluated using RT-qPCR and the gene expression was calculated using the 2^−ΔΔCt^ method, as previously described^49^. Briefly, wild-type *C. albicans* SC5314 was grown overnight in YPD broth at 35 °C and then back-diluted to an OD_600_ of 0.1 in YPD. The cultures were treated with subinhibitory concentrations of ospemifene (0, 1, 10, and 20 µg/ml) and incubated for 3 h at 35 °C. Cells were subsequently collected by centrifugation, RNA was isolated using an Ambion Ribopure yeast kit and cDNA was synthesized using the SuperScript III First-Strand kit (Invitrogen), following the manufacturer’s guidelines. The primers used in this study are provided in Supplementary Table 4. Expression was internally normalized to *ACT1* and compared to the untreated control.

### Caenorhabditis elegans assay

We utilized the *C. elegans* infection model to test whether ospemifene can potentiate the antifungal activity of itraconazole *in vivo*, following previously reported protocols^50,51^. Briefly, synchronized worms [strain AU37 genotype glp-4(bn2) I; sek-1(km4) X] at L4 phase were infected with ~1 × 10^6^ CFU/ml of the fungal suspension for 3 h at room temperature. Infected nematodes were washed at least five times with PBS to remove the non-ingested yeast cells. The infected nematodes were subsequently treated with DMSO (1%), ospemifene (5 µg/ml), itraconazole (1 µg/ml), or a combination of both drugs (all test agents were evaluated in triplicates). Treated worms were incubated at 25 °C for 24 h and were inspected microscopically to confirm viability. *C. elegans* homogenates were prepared by vortexing treated worms with silicon carbide beads for two minutes. The homogenates were serially diluted in PBS and plated over YPD agar plates containing gentamicin (100 g/ml). Plates were incubated for 24 h at 35 °C before CFU per worm was determined.

### Statistical analyses

All experiments were conducted using triplicate samples for each test agent. The statistical analysis was performed using GraphPad Prism 6.0 (Graph Pad Software, La Jolla, CA, USA). The treated groups were compared to control groups using one-way ANOVA, and *P* < 0.05 was considered statistically significant. For Nile red efflux assays, multiple t-tests, using the Holm-Sidak method, were used to assess multiple comparisons between the tested groups.

## Supplementary information

Supplementary Materials.
