# Supplementary material for: Ospemifene displays broad-spectrum synergistic interactions with itraconazole through potent interference with fungal efflux activities
Source: Sci Rep. 2020 Apr 8;10:6089. doi: 10.1038/s41598-020-62976-y (PMC7142066; doi:10.1038/s41598-020-62976-y)
Supplement: Supplementary file 1 — Supplementary Materials. [file 41598_2020_62976_MOESM1_ESM.pdf]

Ospemifene displays broad-spectrum synergistic interactions with itraconazole through potent interference with fungal efflux activities

Hassan E. Eldesouky[1], Ehab A. Salama[1], Tony R. Hazbun [2,3], Abdelrahman S. Mayhoub [4*] and Mohamed N. Seleem [1,5*]

[1]Department of Comparative Pathobiology, College of Veterinary Medicine, Purdue University, West Lafayette, IN 47907, USA

[2]Department of Medicinal Chemistry and Molecular Pharmacology, College of Pharmacy, Purdue University, West Lafayette, Indiana 47907, USA

[3]Bindley Bioscience Center, Purdue University, West Lafayette, Indiana 47906, USA

[4]University of Science and Technology, Nanoscience Program, Zewail City of Science and Technology, October Gardens, 6th of October, Giza 12578, Egypt.

[5]Purdue Institute of Inflammation, Immunology, and Infectious Disease, Purdue University, West Lafayette, IN 47907, USA

*Correspondence:

mseleem@purdue.edu

Supplementary Table 1. Source and description of the fungal strains used in this study.

Fungal Strains	Source	Description
C. albicans SC5314	ATCC	Wild-type strain
C. albicans NR-29448	BEI Resources	A fluconazole-resistant bloodstream isolate (Arizona, USA)
C. albicans NR-29437	BEI Resources	Fluconazole-resistant bloodstream isolate (China)
C. albicans ATCC 26790	ATCC	A fluconazole-resistant strain isolated from a patient with pulmonary candidiasis
C. albicans ATCC MYA-573	ATCC	A fluconazole-resistant bloodstream isolate (from an AIDS patient in Germany)
C. albicans TWO7241	Professor Theodore White (University of Missouri-Kansas City)	A fluconazole-resistant clinical isolate that has increased efflux activity (*MDR1* overexpression) and overexpression of the azole target (*ERG11*)
C. albicans TWO7243		Fluconazole- and itraconazole-resistant clinical isolate that has increased mRNA levels of *CDR1*, *MDR1*, and *ERG11*
C. albicans SCTAC^{1R34A}	Professor David Rogers (University of Tennessee)	A mutant strain having a gain-of-function homozygous mutation in *TAC1*
C. albicans SCMRR^{1R34A}		A mutant strain having a gain-of-function homozygous mutation in *MRR1*
C. glabrata ATCC 66032	ATCC	Not available
C. glabrata ATCC MYA-2950	ATCC	Not available
C. glabrata ATCC 2001	ATCC	A clinical isolate from human intestinal fluid
C. glabrata HM-1123	BEI-Resources	A clinical isolate from the bronchi of a human patient (Missouri, USA)
C. auris 385	CDC	Resistent to fluconazole, itraconazole, voriconazole, and amphotericin B
C. auris 386	CDC	Resistent to fluconazole, voriconazole, and amphotericin B
C. auris 388	CDC	Resistent to fluconazole, itraconazole, voriconazole, and amphotericin B
C. auris 389	CDC	Resistent to fluconazole, itraconazole, voriconazole, and amphotericin B
C. auris 390	CDC	Resistent to fluconazole, itraconazole, and amphotericin B

C. neoformans NR-41291	BEI Resources	A clinical isolate of human cerebrospinal fluid was identified in July 2011 (China)
C. neoformans NR-41295	BEI Resources	A Fluconazole-resistant human cerebrospinal fluid clinical isolate was identified in February 2012 (China)
C. neoformans NR-41298	BEI Resources	A clinical isolate obtained in February 2012 from human cerebrospinal fluid (China)
A. fumigatus NR-35304	BEI-Resources	A clinical isolate identified in 1998 from human sputum tracheal suction in California, USA
A. fumigatus NR-35312	BEI Resources	An environmental isolate was identified in 2002 (Montréal, Québec, Canada)
A. fumigatus NR-35302	BEI Resources	Clinical isolate obtained in 1998 from peritoneal fluid of a human patient in California, USA
S. cerevisiae AD/CaCDR1	Professor Richard D. Cannon (University of Otago, New Zealand)	Recombinant *S. cerevisiae* strain overexpressing *CDR1* from *C. albicans*
S. cerevisiae AD/CaCDR2		Recombinant *S. cerevisiae* strain overexpressing *CDR2* from *C. albicans*
S. cerevisiae AD/CaMDR1		Recombinant *S. cerevisiae* strain overexpressing *MDR1* from *C. albicans*

Supplementary Table 2. Effect of ospemifene-fluconazole (FLC) combination against different fungal strains.

Fungal Strains	MIC (µg/ml)				ΣFICI[1]	Interaction
	FLC		Ospemifene			
	Alone	Combined	Alone	Combined		
C. albicans SC5314	0.125	0.125	> 256	4	1.02	IND
C. albicans NR-29448	256	4		2	0.02	SYN
C. albicans NR-29437	128	4		4	0.05	SYN
C. albicans ATCC MYA 573	128	128		4	1.02	IND
C. albicans TWO7241	32	32		4	1.02	IND
C. albicans TWO7243	64	64		4	1.02	IND
C. albicans SC-TAC1^{G980E}	2	2		4	1.02	IND
C. albicans SC-MRR1^{P683S}	2	2		4	1.02	IND
C. glabrata ATCC 66032	4	4		4	1.02	IND
C. glabrata ATCC MYA-2950	8	8		4	1.02	IND
C. glabrata ATCC 2001	4	4		4	1.02	IND
C. glabrata HM-1123	4	4		4	1.02	IND
C. auris 385	256	256		4	1.02	IND
C. auris 386	256	256		4	1.02	IND
C. auris 388	256	256		4	1.02	IND
C. auris 389	256	256		4	1.02	IND
C. auris 390	256	256		4	1.02	IND
C. neoformans NR-41291	8	1		0.5	0.13	SYN
C. neoformans NR-41295	8	4		1	0.50	SYN
C. neoformans NR-41298	2	0.5		0.5	0.25	SYN
A. fumigatus NR-35304	> 256	> 256		4	1.02	IND
A. fumigatus NR-35312	> 256	> 256		4	1.02	IND
A. fumigatus NR-35302	> 256	> 256		4	1.02	IND

[1] ΣFICI (fractional inhibitory concentration index) values, rounded to the nearest two decimal places, were used to measure the interaction between the tested combinations. ΣFICI interpretation corresponded to the following definitions: synergism (SYN), ΣFICI ≤ 0.50; additivity (ADD), ΣFICI > 0.50 and ≤ 1; and indifference (IND), ΣFICI > 1 and ≤ 4.

Supplementary Table 3. Effect of ospemifene-voriconazole (VRC) combination against different fungal strains.

**Fungal Strains**	**MIC (µg/ml)**				**ΣFICI***	**Interaction**
	**VRC**		**Ospemifene**			
	**Alone**	**Combined**	**Alone**	**Combined**		
C. albicans SC5314	0.031	0.031		4	1.02	IND
C. albicans NR-29448	4	0.125		8	0.06	SYN
C. albicans NR-29437	0.125	0.015		4	0.14	SYN
C. albicans ATCC MYA 573	0.25	0.25		4	1.02	IND
C. albicans TWO7241	0.125	0.062		1	0.50	SYN
C. albicans TWO7243	0.50	0.50		4	1.02	IND
C. albicans SCTAC1R34A	0.062	0.062		4	1.02	IND
C. albicans SCMRR1R34A	0.015	0.015		2	1.01	IND
C. glabrata ATCC 66032	0.125	0.125		2	1.02	IND
C. glabrata ATCC MYA-2950	0.125	0.125		2	1.01	IND
C. glabrata ATCC 2001	0.062	0.062		2	1.01	IND
C. glabrata HM-1123	0.062	0.062	> 256	2	1.01	IND
C. auris 385	2	2		4	1.02	IND
C. auris 386	1	1		4	1.02	IND
C. auris 388	1	1		4	1.02	IND
C. auris 389	2	2		4	1.02	IND
C. auris 390	0.50	0.50		4	1.02	IND
C. neoformans NR-41291	0.125	0.062		0.5	0.50	SYN
C. neoformans NR-41295	0.25	0.062		0.5	0.25	SYN
C. neoformans NR-41298	0.031	0.015		0.5	0.50	SYN
A. fumigatus NR-35304	0.125	0.125		4	1.02	IND
A. fumigatus NR-35312	0.125	0.125		4	1.02	IND
A. fumigatus NR-35302	0.125	0.125		4	1.02	IND

[1] ΣFICI (fractional inhibitory concentration index) values, rounded to the nearest two decimal places, were used to measure the interaction between the tested combinations. ΣFICI interpretation corresponded to the following definitions: synergism (SYN), ΣFICI ≤ 0.50; additivity (ADD), ΣFICI > 0.50 and ≤ 1; and indifference (IND), ΣFICI > 1 and ≤ 4.

Supplementary Table 4. Primers used in this study.

Gene	Protein	GeneBank accession no.	Primer	Sequence
ACT1	Actin	XM_019475182	Forward<br>Reverse	TTGGTGATGAAGCCCAATCC<br>CATATCGTCCCAGTTGGAAACA
MDR1	Major facilitator superfamily transporter	XM_714072	Forward<br>Reverse	TTACCTGAAACTTTTGGCAAAACA<br>ACTTGTGATTCTGTCGTTACCG
CDR1	ABC transporters CDR1	XM_718116	Forward<br>Reverse	TTTAGCCAGAACTTTCACTCATGATT<br>TATTTATTTCTTCATGTTCATATGGATTGA
CDR2	ABC transporters CDR2	XM_718076	Forward<br>Reverse	GGTATTGGCTGGTCCTAATGTGA<br>GCTTGAATCAAATAAGTGAATGGATTAC

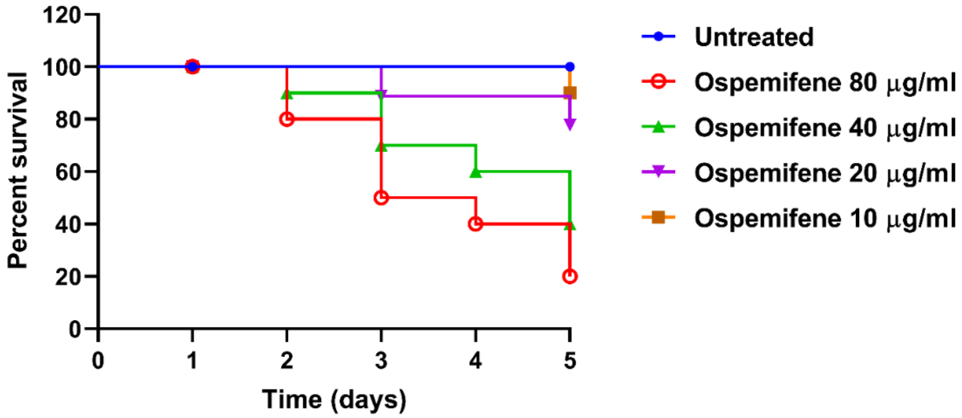

Supplementary Figure 1. Toxicity assessment of ospemifene in *C. elegans*. Adult (L4-stage) nematodes were exposed to ospemifene at four different concentrations (80, 40, 20, and 10 µg/ml) and viability was recorded for five consecutive days.

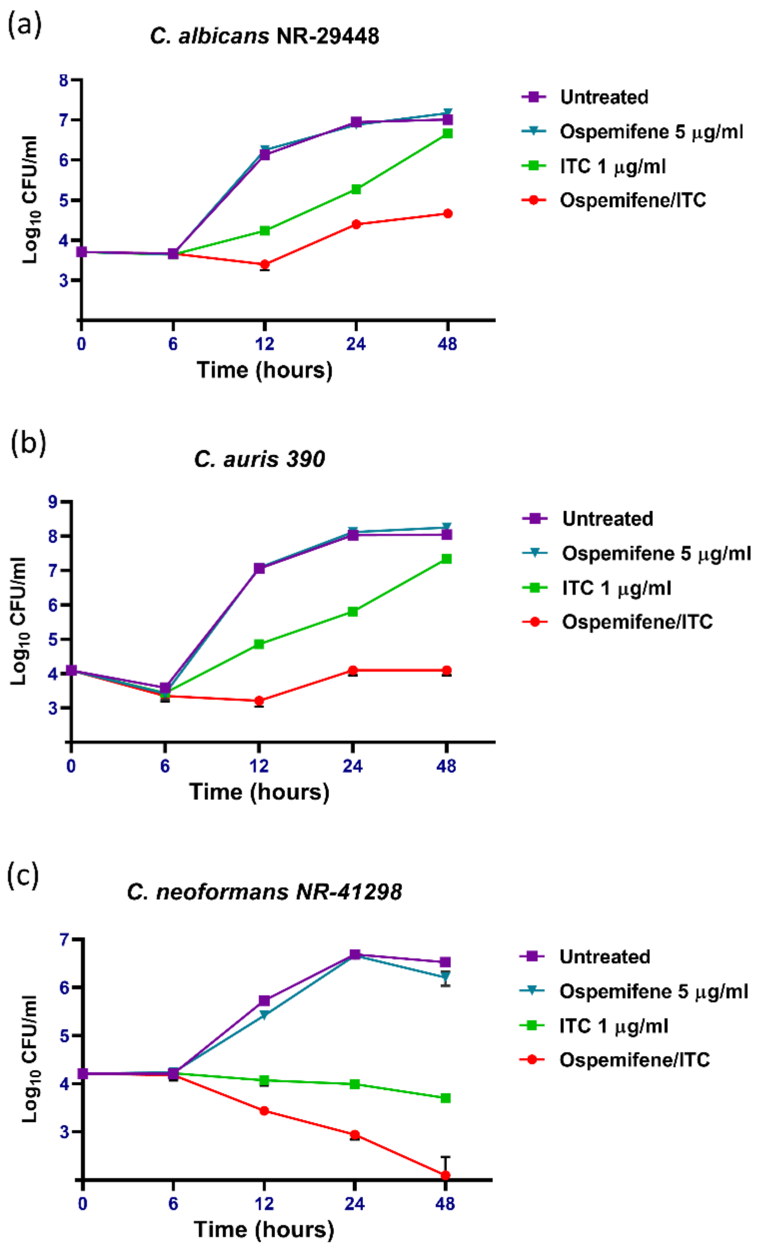

Supplementary Figure 2. Time-kill analysis of ospemifene (5 µg/ml), itraconazole (1 µg/ml), or a combination of both. Test agents were evaluated against (**a**) *C. albicans* NR-29448, (**b**) *Candida auris* 390, and (**c**) *C. neoformans* NR-41298 over a 48-hour incubation period at 35 °C. DMSO served as a negative untreated control. Error bars represent standard deviation values.

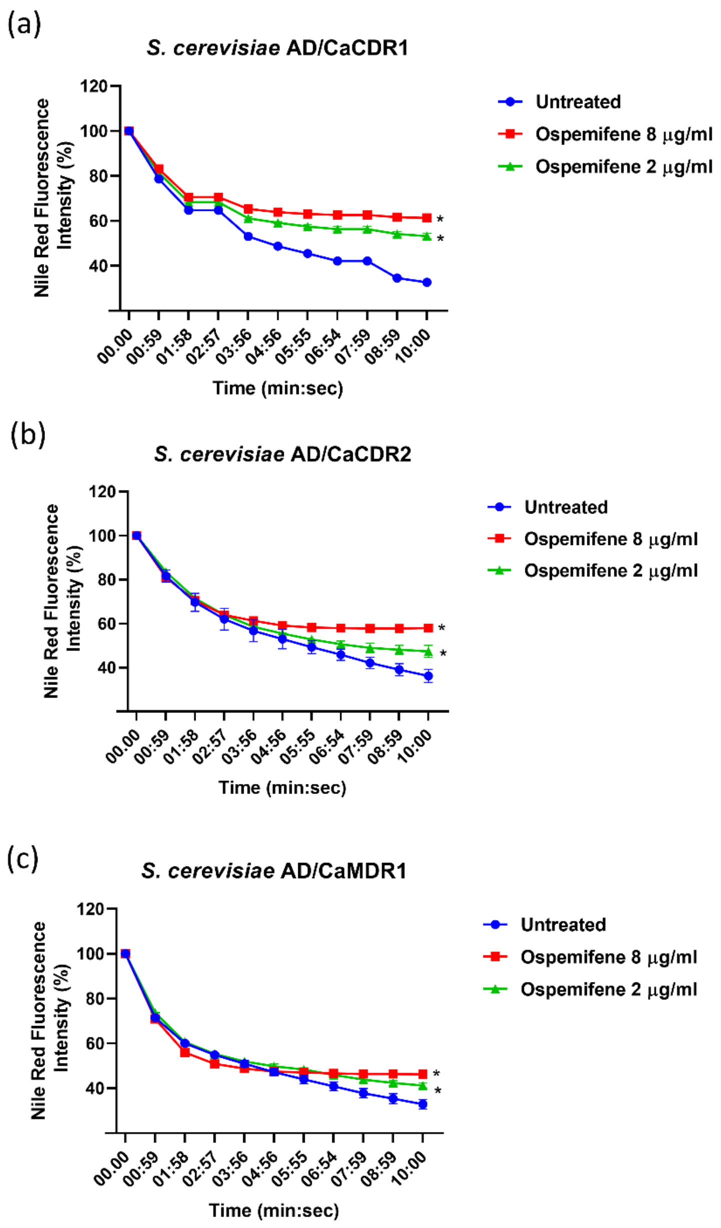

Supplementary Figure 3. Effect of ospemifene on Nile red efflux by recombinant *Saccharomyces cerevisiae* strains. Effect of ospemifene on Nile red efflux was evaluated against recombinant *S. cerevisiae* strains (a) Recombinant *S. cerevisiae* AD/CaCDR1 overexpressing *C. albicans CDR1* (b) Recombinant *S. cerevisiae* AD/CaCDR2 overexpressing *C. albicans CDR2*, and (c) Recombinant *S. cerevisiae* AD/CaMDR1 overexpressing *C. albicans MDR1*. Energy-depleted cells were loaded with Nile red (7.5 µM) and were treated with ospemifene (2, and 8 µg/ml). Efflux was initiated by adding glucose (40 mM) to all treatment groups. The Nile red fluorescence intensity was then monitored over 10 minutes and is expressed as the percentage of change in the fluorescence intensity. (*) indicates a significant statistical difference in the fluorescence intensity of the treated cells relative to the untreated control ($P < 0.05$), as determined by multiple t-tests using the Holm-Sidak statistical method for multiple comparisons.

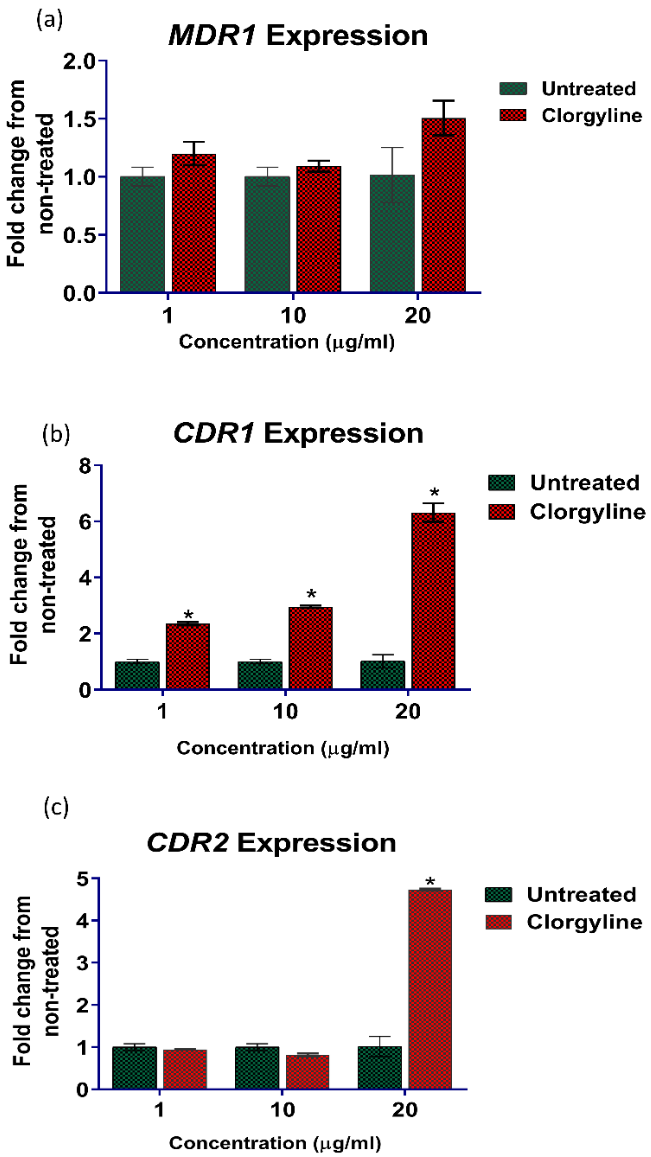

Supplementary Figure 4. Effect of clorgyline on the expression of azole resistance-related efflux genes a) *MDR1*, b) *CDR1*, and c) *CDR2*. *C. albicans* SC5314 was treated with either DMSO or ospemifene (1, 10, 20 µg/ml) for 3 h in RPMI 1640 and harvested. The expression of *CDR1*, *CDR2*, and *MDR1* was determined by quantitative RT-PCR. Bars show the mean fold change for ospemifene-treated cells relative to untreated cells, and error bars indicate standard deviations of three biological replicates. * indicates a significant difference between clorgyline treatments relative to the untreated control ($P < 0.05$), as determined by multiple t-tests using the Holm-Sidak statistical method for multiple comparisons.
